# Supplementary material for: Epistaxis With Warfarin Coagulopathy: An Adult Simulation Case for Residents
Source: MedEdPORTAL. 2020 Jun 26;16:10916. doi: 10.15766/mep_2374-8265.10916 (PMC7331959; doi:10.15766/mep_2374-8265.10916)
Supplement: Supplementary file 1 — Simulation Case.docxSimulation Images.pptxPrebrief.docxDebriefing Materials.docxCritical Action Checklist.docxLearner Evaluation Form.docxHandout and Video Review.docx [file mep_2374-8265.10916-s001.zip › A. Simulation Case.docx]

| **Appendix A: MedEdPORTAL Simulation Case**  **SIMULATION CASE TITLE:** "Epistaxis with Warfarin Coagulopathy: An Adult Simulation Case for Residents"  **AUTHOR: Jonathon Deibel, MD** | |
| --- | --- |
| **PATIENT NAME: Henry**  **PATIENT AGE: 82-year-old male**  **CHIEF COMPLAINT: Nose bleed for four hours** | |
|  | |
| **Brief narrative description of case** | Pt arrived via triage for nose bleeding that has not stopped for the last 4 hours. |
| **Primary Learning Objectives** | 1. Demonstrate appropriate management of a patient presenting with active epistaxis including compression and packing or cautery [Patient Care and Procedural Skills]. 2. Treat blood-loss anemia and coagulopathy appropriately [Medical Knowledge]. 3. Demonstrate effective leadership of a treatment team in the emergency department setting [Interpersonal and Communication Skills]. |
| **Critical Actions** | 1. Immediately address epistaxis with compression, follow with nasal packing or cautery 2. Treat acute blood-loss anemia and shock with blood products 3. Recognize mild hypertension and choose not to treat with antihypertensive medications in the setting of acute blood loss anemia 4. Treat warfarin-induced coagulopathy with appropriate reversal agent 5. Recognize airway obstruction and treat with suctioning 6. Communicate clearly with consultant(s) 7. Direct appropriate ICU admission |
| **Learner Preparation** | - Tour of sim lab. - Introduction about the high-fidelity simulation for learners. - Triage report with chief complaint and vital signs. - Prebrief. |

| Initial Presentation | | | |
| --- | --- | --- | --- |
| **Initial vital signs** | VS: Temp. 37.1 ◦C (98.8 ◦F), HR 102, RR 24, BP 188/98, O2 sat 96% RA. | | |
| **Overall Appearance** | Elderly, mildly anxious, blood dripping from nose | | |
| **Actors and roles in the room at case start** | - Patient voice is male - ED simulated nurse can start IV and assist as requested - ENT:  available by phone for consultation - No family member available | | |
| HPI | This is an 82-year-old male who presents with 4 hours of nose bleeding. He has tried pinching the nose, but bleeding only improved minimally. He has no history of similar nose bleeding. He is on warfarin for history or atrial fibrillation. He is beginning to feel anxious with the continued bleeding. He is feeling somewhat presyncopal. He denies shortness of breath or chest pain. | | |
| **Past Medical/Surgical History** | **Medications** | **Allergies** | **Family/social History** |
| - Hypertension - Atrial fibrillation - Hyperlipidemia - Coronary artery disease | - Hydrochlorothiazide 25mg daily - Atorvastatin 50mg daily - Aspirin 81mg daily - Warfarin 5mg daily | - No known allergies | 1 PPD tobacco for 20 years (quit 45 years ago), Social EtOH, no illicit substances including no cocaine |
| **Physical Examination** | | | |
| **General** | Elderly male, anxious, actively bleeding from nose. | | |
| **HEENT** | Normocephalic and atraumatic, conjunctivae and EOM are normal. Pupils are equal, round and reactive to light. Active bleeding from the right nostril. | | |
| **Neck** | Normal range of motion. Neck supple. No JVD. | | |
| **Lungs** | Tachypneic. No wheezes or rales. | | |
| **Cardiovascular** | Tachycardic, irregular rhythm. Pulses are palpable and equal bilaterally. | | |
| **Abdomen** | Soft. He exhibits no distension. Bowel sounds are normal. There is no tenderness. There is no guarding. | | |
| **Neurological** | He is alert and oriented to person, place, and time. She has normal strength. No cranial nerve deficit or sensory deficit. | | |
| **Skin** | No rash noted. | | |
| **GU** | No rash, Penis normal. Circumcised. | | |
| **Psychiatric** | Anxious. | | |

| Instructor Notes - Changes and CASE Branch Points*.* | | |
| --- | --- | --- |
| **Intervention / Time point** | **Change in Case** | **Additional Information** |
| Initial assessment | No change in vital signs. Active bleeding from nose which continues with manual compression or placement of a clip. | Allow learner 4 minutes to obtain focused history and physical examination. |
| Anytime | If antihypertensive agent is given for moderate hypertension patient will decompensate. Decrease in blood pressure to 90/54, heart rate increases to 120, respiratory rate increases to 30, SpO2 drops to 92% on room air. Vital signs will improve if blood is given. |  |
| 4 minutes | Vital signs change somewhat as patient continues to bleed from the nose. BP 182/94, HR 106, RR: 22, Sp02 95%. |  |
| 10 minutes | If PRBC and reversal agent are not given, vital signs worsen to blood pressure 80/50, heart rate 120, respiratory rate 30, SpO2 92% on room air. If PRBC and reversal agent are given, vital signs improve to blood pressure 162/92, heart rate 88, respiratory rate 22, SpO2 92% on room air. |  |
| Anytime | As learner performs the nasal packing a blood clot is pushed into the pharynx causing the patient to choke. If suctioning is performed in the mouth or if the patient is intubated he will stop choking. If these interventions are not performed in 1 minute the patient will have a PEA arrest until suctioning is performed or the patient is intubated. | Learners should recognize that the patient was unable to blow the blood clot from the nose and that the clot is obstructing the airway. Learners should initiate prompt suctioning. |
| 13 minutes | Patient vital signs will stabilize after receiving blood products. |  |
| Calls made for disposition and consultation | Final disposition should be admission to the ICU with ENT and possibly cardiology consultation. | ENT or intensivist can prompt for critical actions not yet performed. |

**PICTORAL FLOW DIAGRAM**


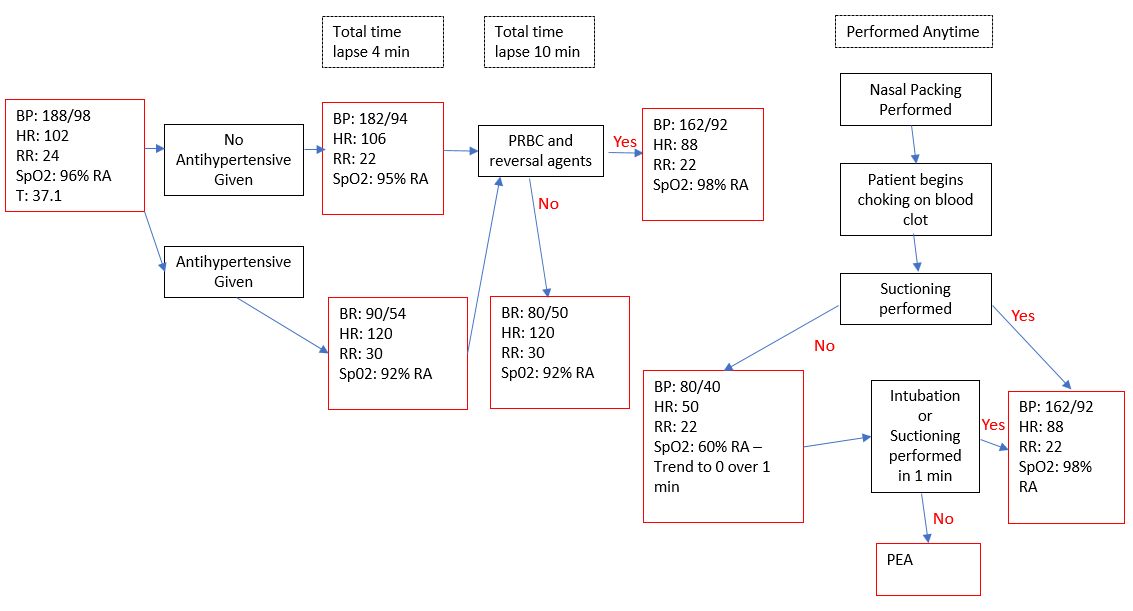


| Environment and Props | |
| --- | --- |
| Simulator Set Up | Male, talking. Epistaxis task trainer. Active bleeding from nose. |
| Equipment available | Cardiac monitor, IV, crash cart, O2 (Nasal cannula, NRB), airway equipment, suction, intranasal packing, nasal clamps. |
| Medications | ACLS medications, common antihypertensives (Hydralazine, Labetalol, clonidine), normal saline 2L, PRBCs, vitamin K, prothrombin complex concentrate (PCC), fresh frozen plasma (FFP), intranasal tranexamic acid, oxymetazoline (Afrin), adrenaline, silver nitrate. |

**Ideal Scenario Flow**

The patient arrives awake, active bleeding from nose. He is anxious and moderately hypertensive. Learners may choose to inappropriately treat with an antihypertensive medication. If an antihypertensive is given the patient will become hypotensive. Hypotension will improve with IV hydration or packed red blood cells. The team should order blood work and recognize the potential need for blood products and coagulopathy reversal. Type and screening should be performed with 2-4 units of packed red blood cells crossed. Laboratory results will demonstrate moderate anemia and coagulopathy. Learner should ask for uncross-matched blood if crossed blood is not immediately available. If packed red blood cells and a reversal agent are not given in a timely manner the patient becomes hypotensive. If packed red blood cells are given the hypotension will resolve. Bleeding will not stop until nasal packing is performed. If the learner prompts the patient to blow his nose prior to packing the patient will not be able to blow his nose with significant force enough to remove a large blood clot. The nose bleeding will stop with posterior packing. Anterior packing will not stop the bleeding. The patient will immediately begin choking as a large blood clot is pushed into the pharynx with nasal packing. The patient will quickly and progressively become hypoxic. Team can suction the clot from the posterior pharynx and vital signs will improve. They may choose to intubate, and this will also improve vital signs. If patient is not suctioned or intubated within 1 minute the patient will decompensate to PEA. Suctioning or intubation will return patient to sinus rhythm with a pulse. The case is complete after the team admits the patient to the ICU and possibly consults the ENT physician.

**Anticipated Management Mistakes**

The most common and consequential learner errors are listed here along with a discussion of corrective measures.

- Failure to promptly address active bleeding. The learners may not initially address the active bleeding. The patient may bring the continued bleeding to the learners’ attention. The simulated nurse may prompt learners to place a clip or have the patient pinch the nose.
- Failure in mistakenly treating the patient’s moderate hypertension. Learners may choose to give an antihypertensive medication. They are likely but wrongfully assuming the moderate hypertension is contributing to the patient’s continued bleeding. The learners should be allowed to make this error. The patient will quickly become hypotensive and not improve until given blood products and have the coagulopathy reversed.
- Failure to order appropriate blood tests. When ordering tests, learners may forget to order a blood type, screen and cross. They may not anticipate the need for blood products. Learners are likely to be aware that most patients with epistaxis do not require blood work. They may also not order a coagulation panel possibly because of an incomplete medication history. The testing results should be withheld until the orders are placed.
- Failure to have the patient blow his nose prior to placement of the nasal packing. Learners should follow a step-wise approach to the treatment of epistaxis. This including having the patient blow his nose prior to placing packing. This clears the nose of blood clots and allows for easier placement of packing. Failure to do this in general may lead to pushing a blood clot into the pharynx. In this case, the patient is unable to forcefully blow his nose and will choke on a blood clot regardless of the learner’s attempts to have the patient blow his nose. The facilitated debrief provides an opportunity to discuss this potential complication of placing the nasal packing.
