# Supplementary material for: Epistaxis With Warfarin Coagulopathy: An Adult Simulation Case for Residents
Source: MedEdPORTAL. 2020 Jun 26;16:10916. doi: 10.15766/mep_2374-8265.10916 (PMC7331959; doi:10.15766/mep_2374-8265.10916)
Supplement: Supplementary file 1 — Simulation Case.docxSimulation Images.pptxPrebrief.docxDebriefing Materials.docxCritical Action Checklist.docxLearner Evaluation Form.docxHandout and Video Review.docx [file mep_2374-8265.10916-s001.zip › B. Simulation Images.pptx]

## Slide 1
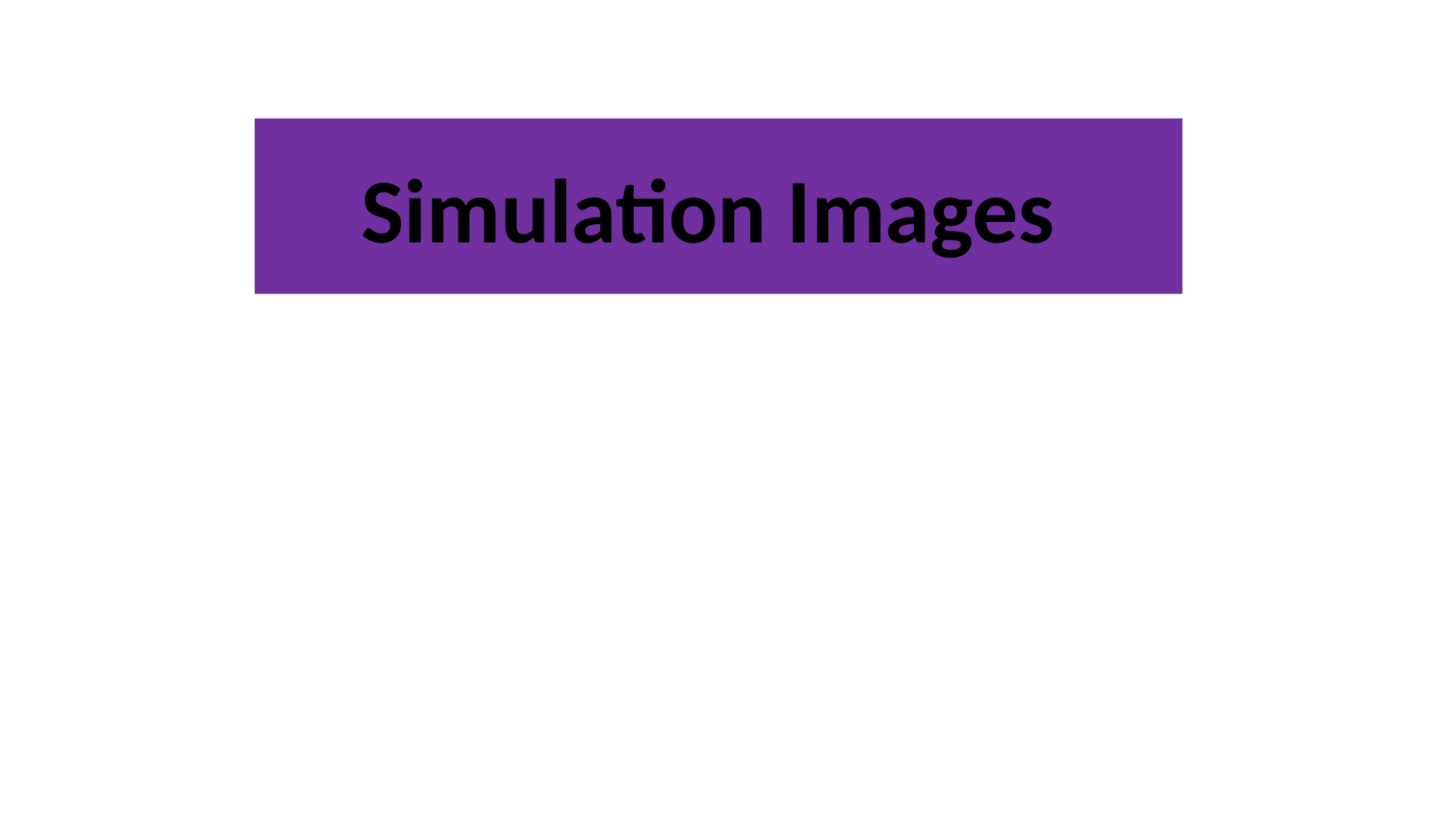

# Simulation Images

## Slide 2
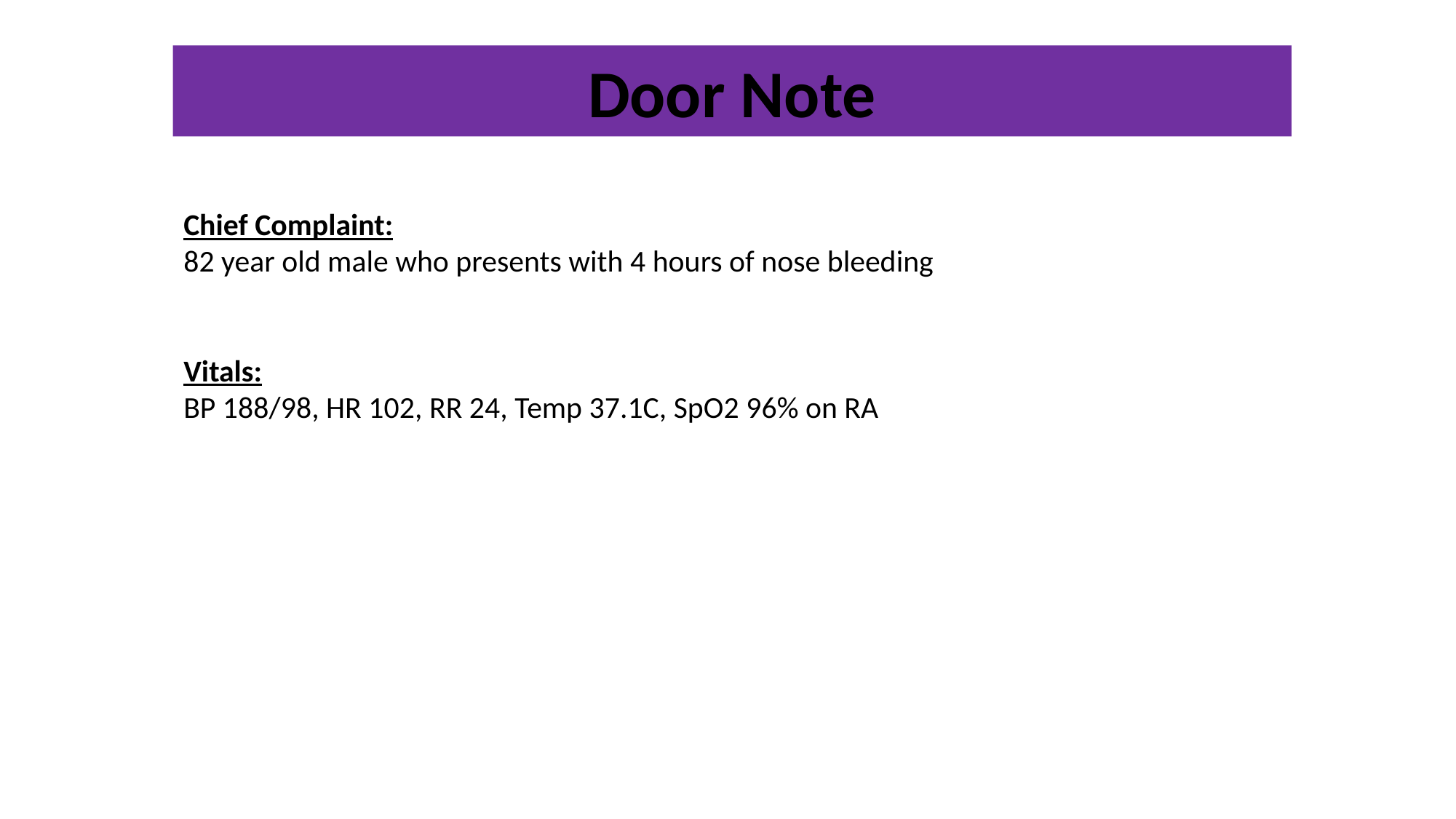

# Door Note
Chief Complaint:
82 year old male who presents with 4 hours of nose bleeding
Vitals:
BP 188/98, HR 102, RR 24, Temp 37.1C, SpO2 96% on RA

## Slide 3
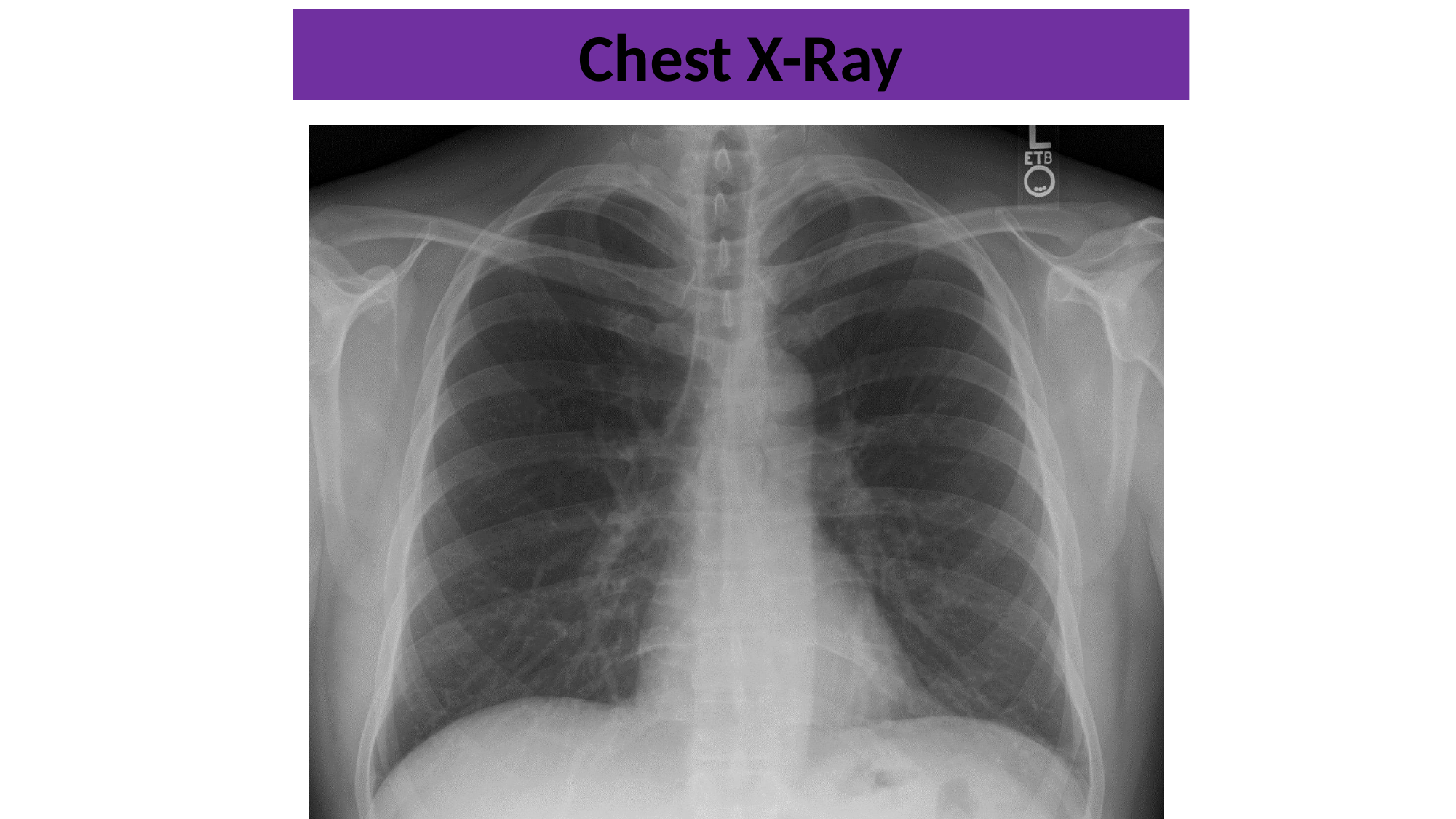

Chest X-Ray

## Slide 4
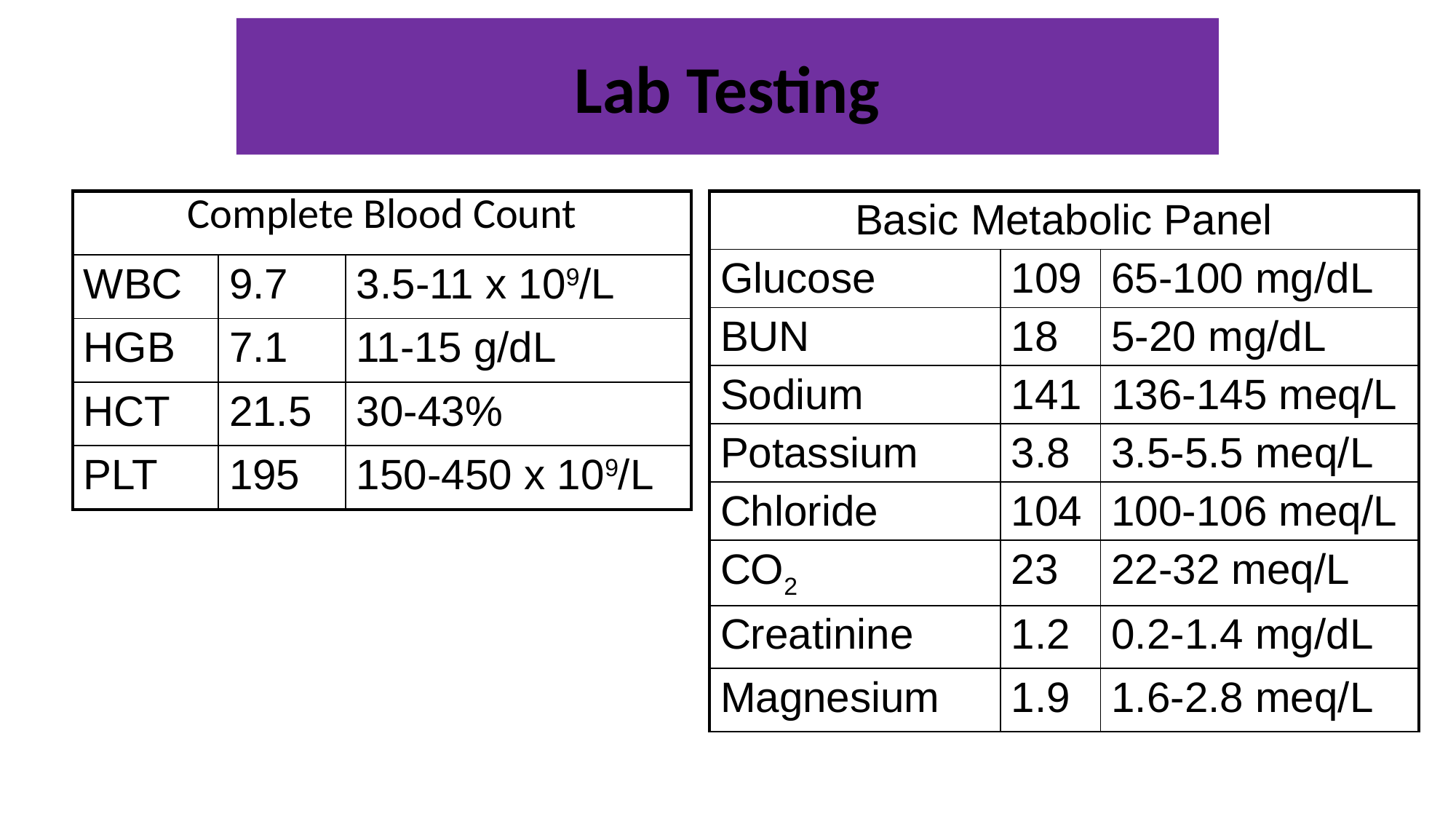

# Lab Testing
| Complete Blood Count | | |
| --- | --- | --- |
| WBC | 9.7 | 3.5-11 x 109/L |
| HGB | 7.1 | 11-15 g/dL |
| HCT | 21.5 | 30-43% |
| PLT | 195 | 150-450 x 109/L |
| Basic Metabolic Panel | | |
| --- | --- | --- |
| Glucose | 109 | 65-100 mg/dL |
| BUN | 18 | 5-20 mg/dL |
| Sodium | 141 | 136-145 meq/L |
| Potassium | 3.8 | 3.5-5.5 meq/L |
| Chloride | 104 | 100-106 meq/L |
| CO2 | 23 | 22-32 meq/L |
| Creatinine | 1.2 | 0.2-1.4 mg/dL |
| Magnesium | 1.9 | 1.6-2.8 meq/L |

## Slide 5
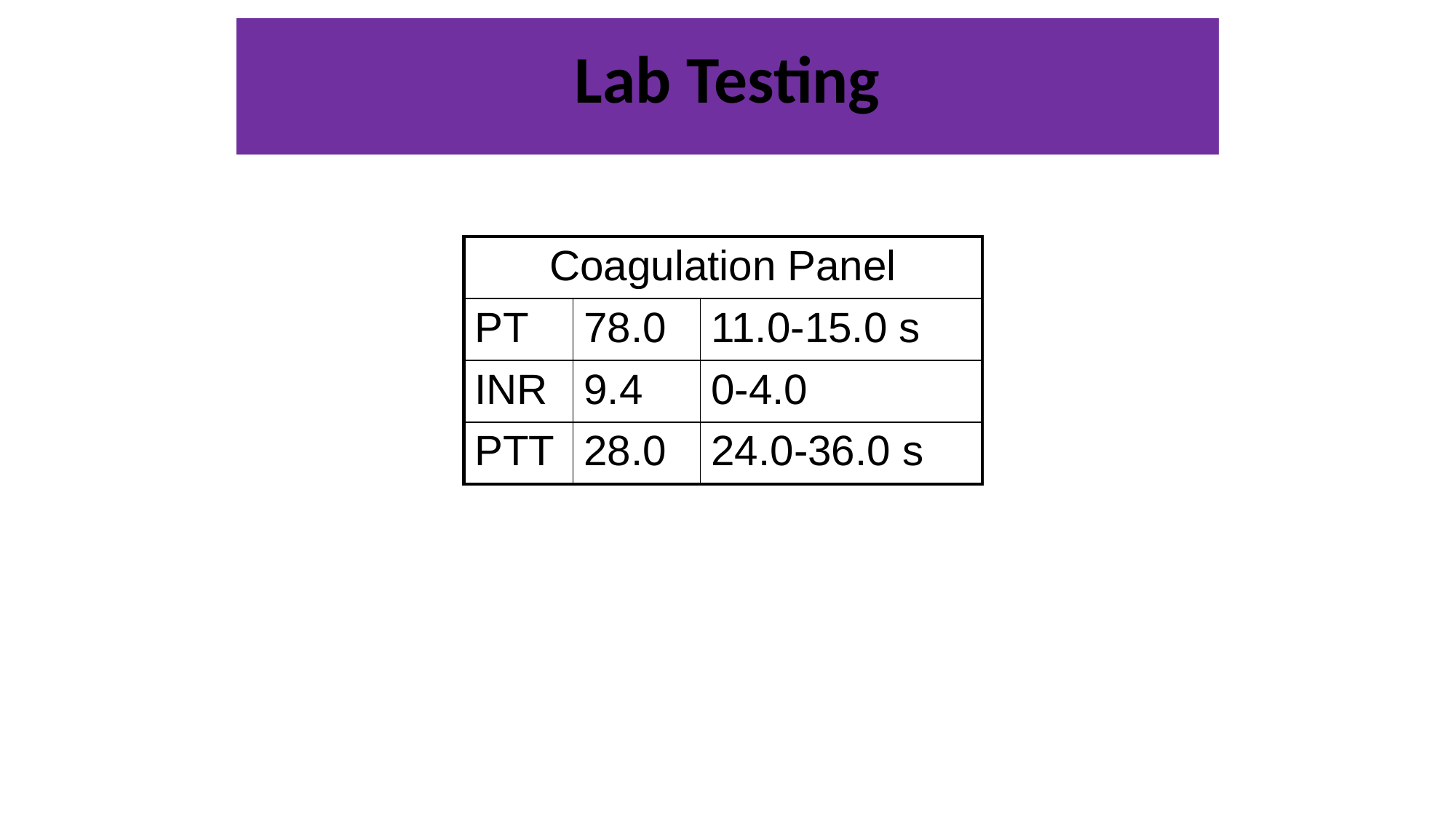

Lab Testing
| Coagulation Panel | | |
| --- | --- | --- |
| PT | 78.0 | 11.0-15.0 s |
| INR | 9.4 | 0-4.0 |
| PTT | 28.0 | 24.0-36.0 s |

## Slide 6
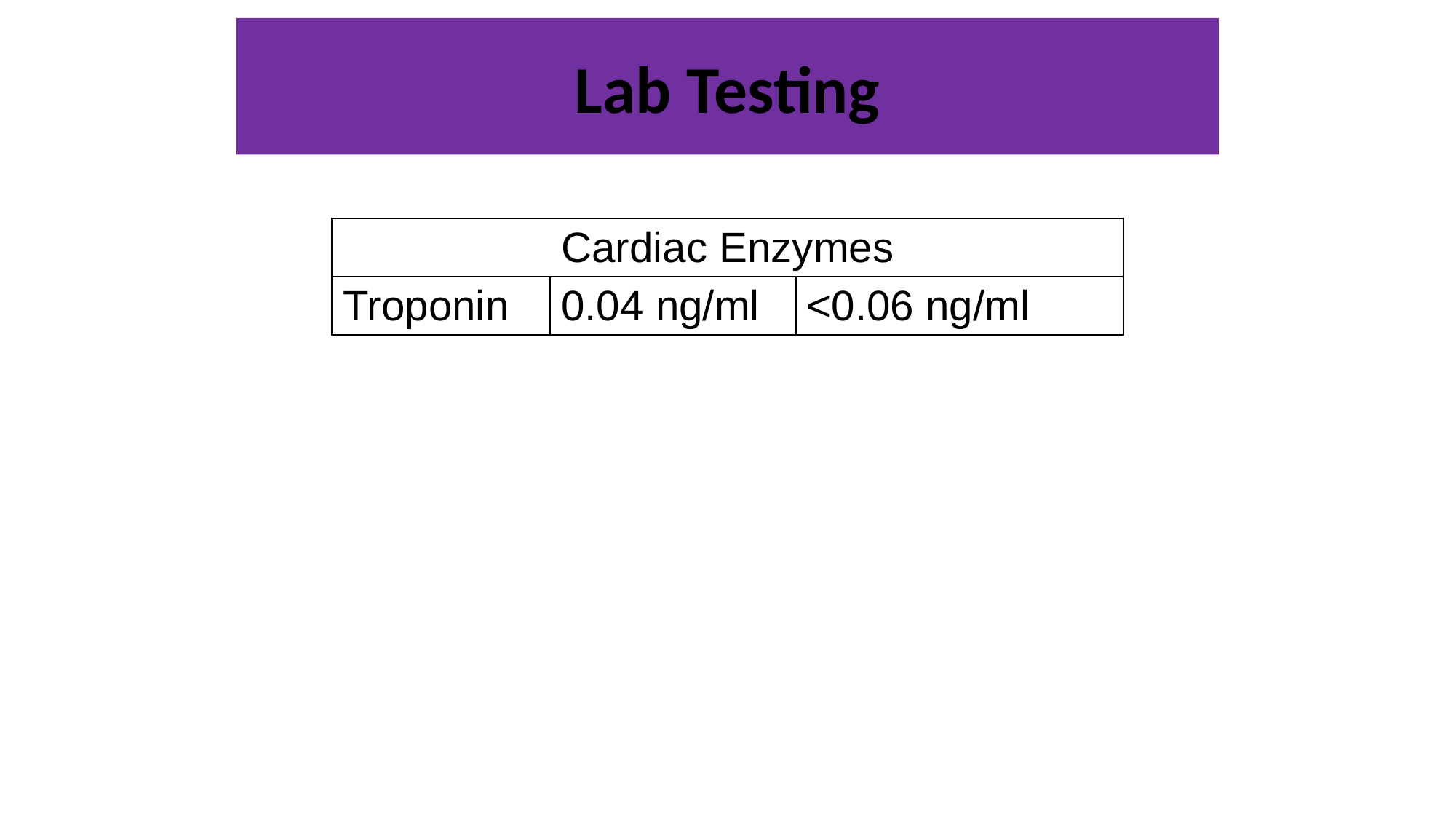

Lab Testing
| Cardiac Enzymes | | |
| --- | --- | --- |
| Troponin | 0.04 ng/ml | <0.06 ng/ml |

## Slide 7
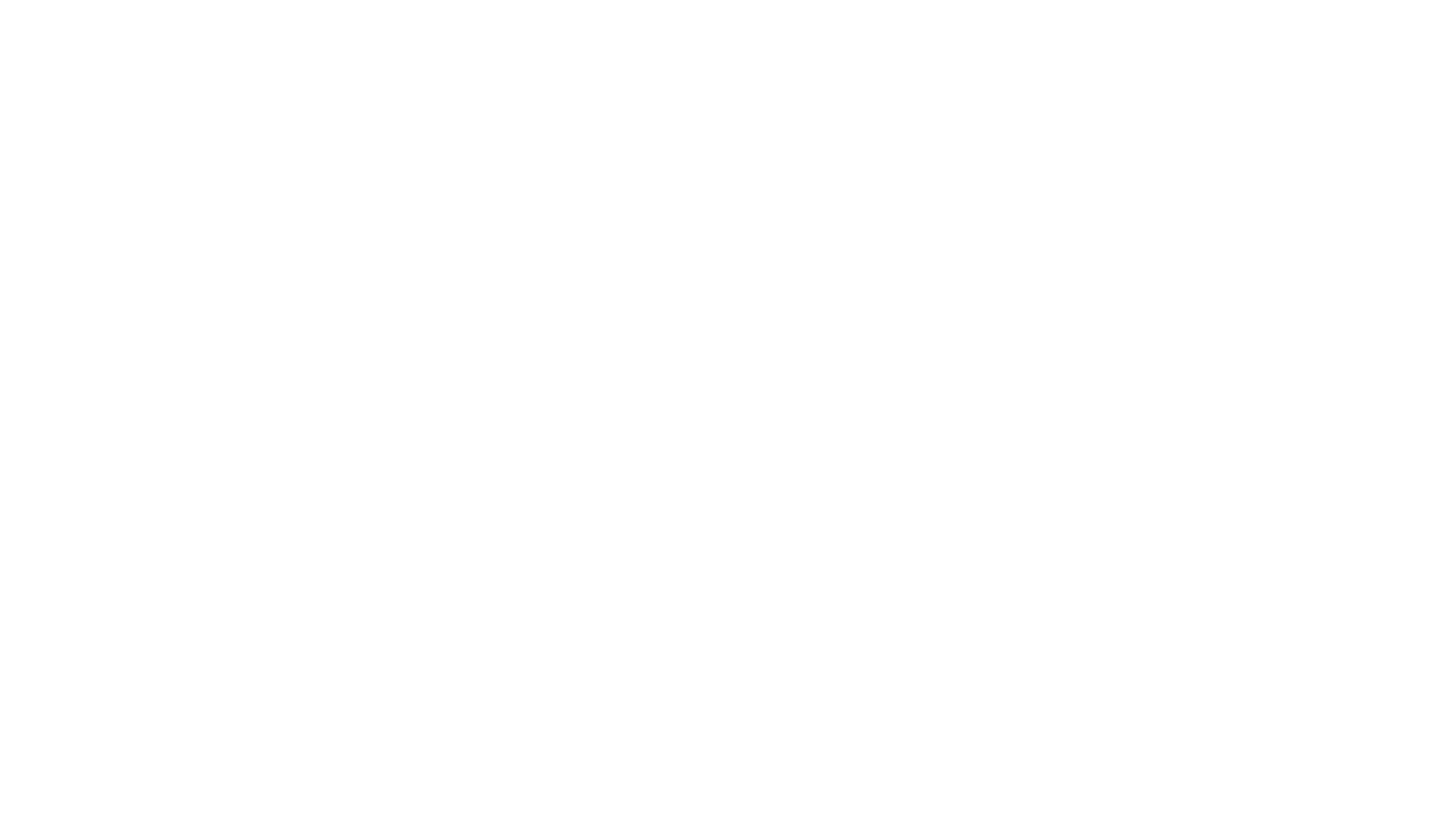

#

## Slide 8
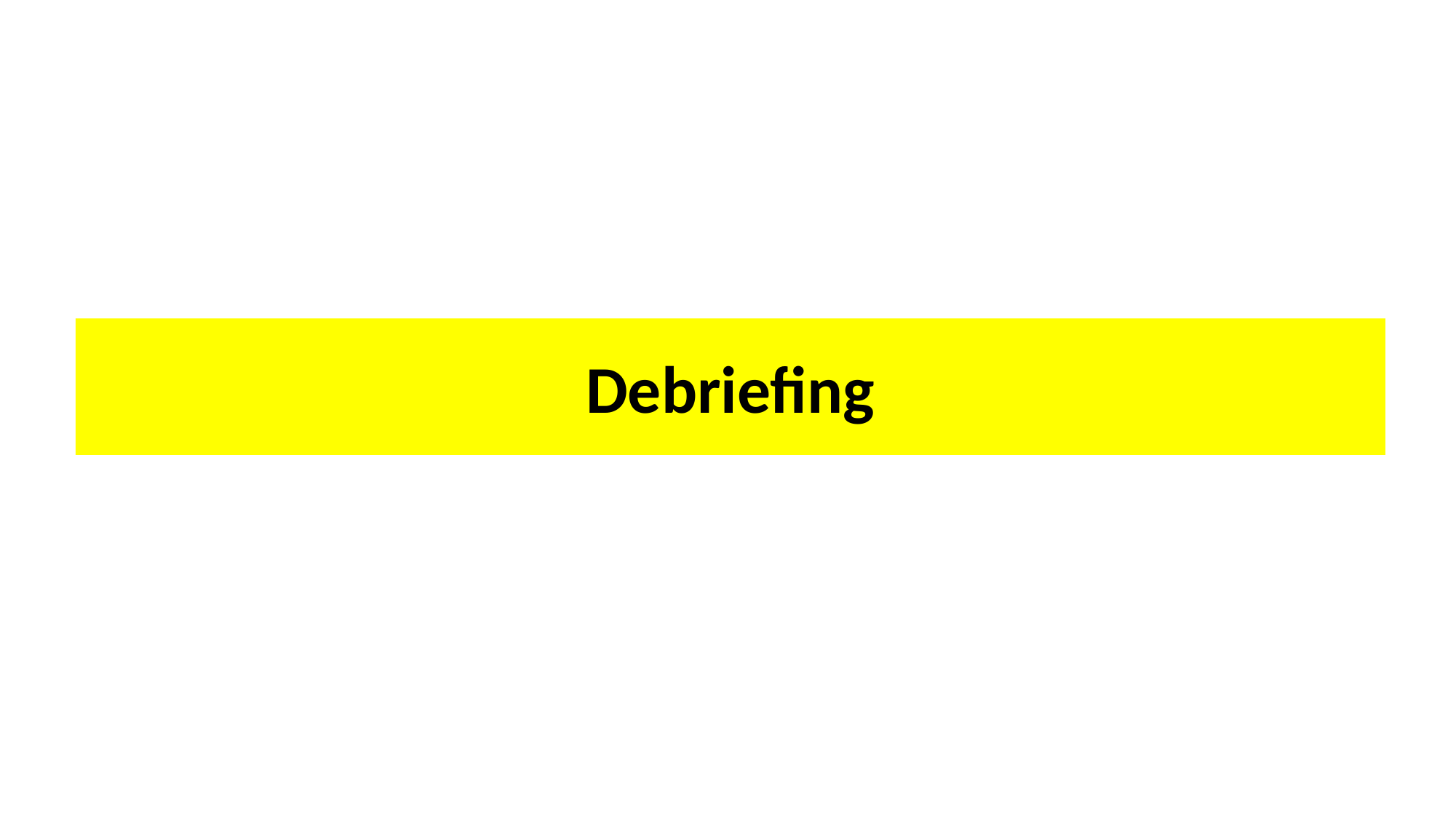

# Debriefing

## Slide 9
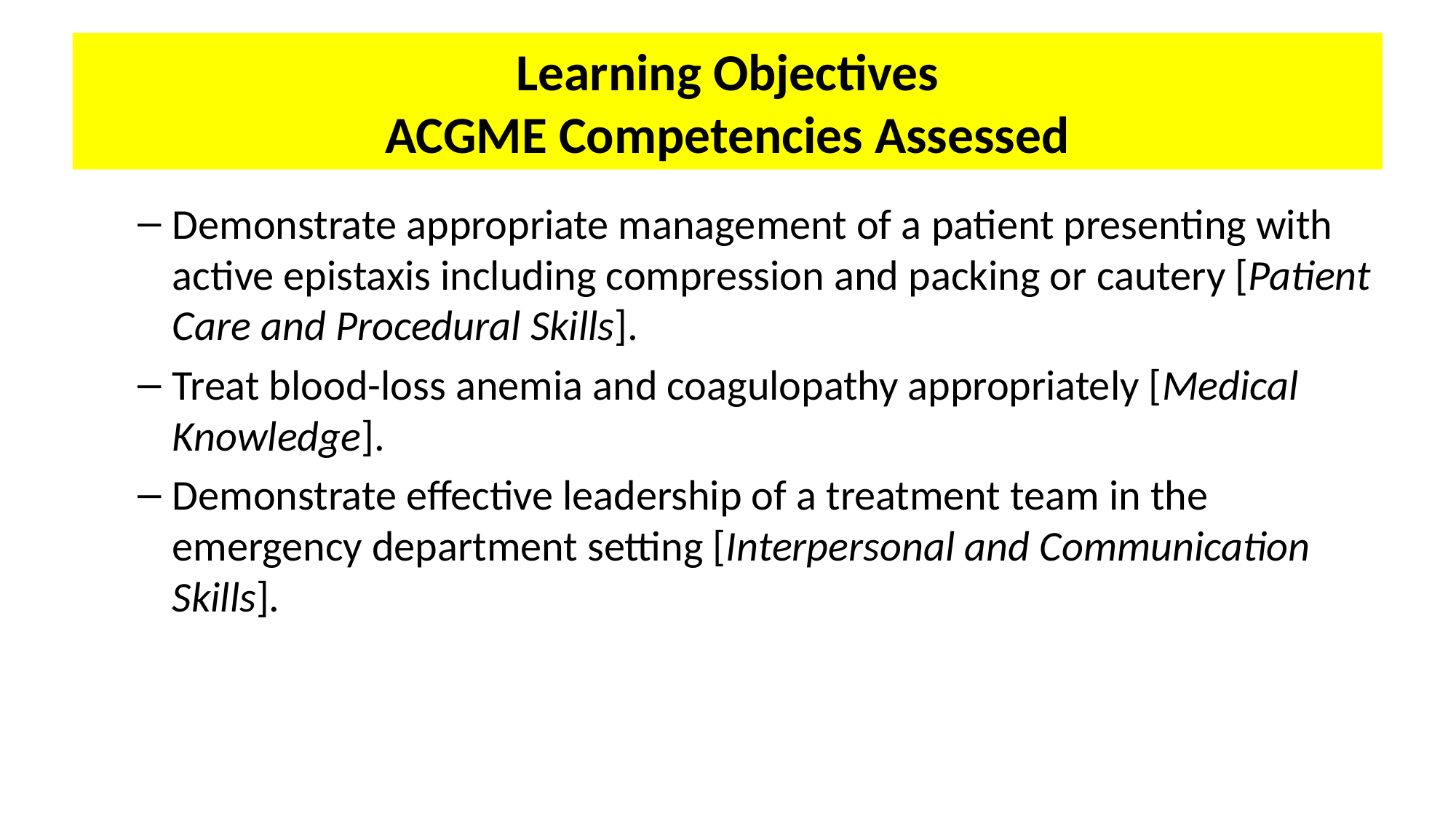

# Learning Objectives ACGME Competencies Assessed
Demonstrate appropriate management of a patient presenting with active epistaxis including compression and packing or cautery [Patient Care and Procedural Skills].
Treat blood-loss anemia and coagulopathy appropriately [Medical Knowledge].
Demonstrate effective leadership of a treatment team in the emergency department setting [Interpersonal and Communication Skills].

## Slide 10
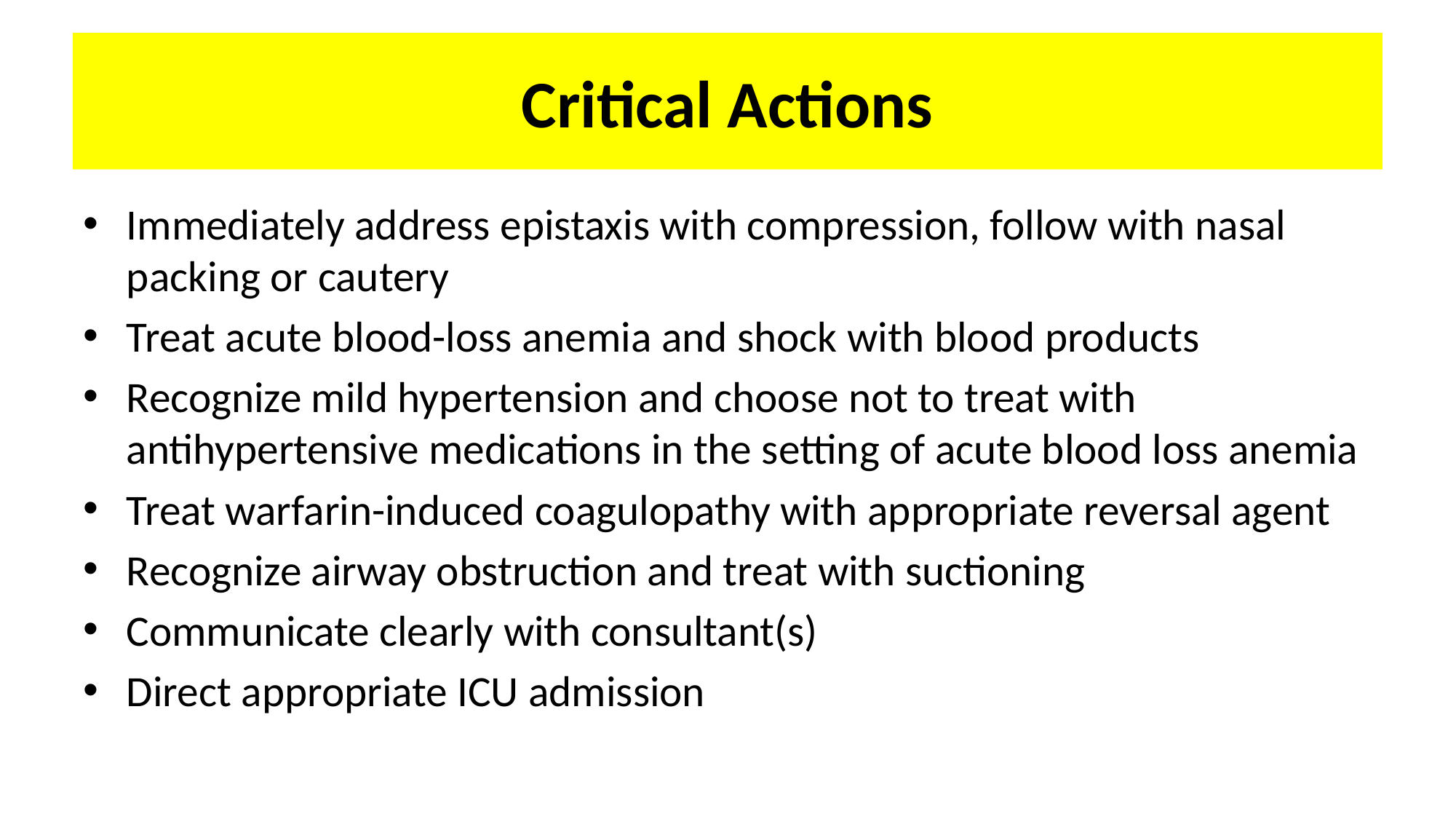

# Critical Actions
Immediately address epistaxis with compression, follow with nasal packing or cautery
Treat acute blood-loss anemia and shock with blood products
Recognize mild hypertension and choose not to treat with antihypertensive medications in the setting of acute blood loss anemia
Treat warfarin-induced coagulopathy with appropriate reversal agent
Recognize airway obstruction and treat with suctioning
Communicate clearly with consultant(s)
Direct appropriate ICU admission
