# Supplementary material for: Epistaxis With Warfarin Coagulopathy: An Adult Simulation Case for Residents
Source: MedEdPORTAL. 2020 Jun 26;16:10916. doi: 10.15766/mep_2374-8265.10916 (PMC7331959; doi:10.15766/mep_2374-8265.10916)
Supplement: Supplementary file 1 — Simulation Case.docxSimulation Images.pptxPrebrief.docxDebriefing Materials.docxCritical Action Checklist.docxLearner Evaluation Form.docxHandout and Video Review.docx [file mep_2374-8265.10916-s001.zip › C. Prebrief.docx]

**Epistaxis with Warfarin Coagulopathy**

**Prebrief**

The prebrief is meant to be sent out several days before the simulation case. The purpose is to guide learners to focus on certain aspects of their resuscitation, communication and management skills. It generally does NOT give clues as to what the case will be. The learners will have the expectation that the specific focus areas included in the prebrief will be discussed in the debrief. The focus of the prebrief follows The Accreditation Council for Graduate Medical Education’s (ACGME) milestones for Emergency Medicine. The focus can be altered for other specialty milestones.

Please be prepared to participate in the upcoming simulation case. Please pay attention to the following ACGME milestones as you prepare for participation in the case. We will discuss each of these as part of the debrief.

1. Emergency Stabilization (PC1): Prioritizes critical initial stabilization actions in the resuscitation of a critically ill or injured patient and reassesses after implementing a stabilizing intervention
2. Performance of Focused Physical Exam (PC2): Performs and communicates a focused history and physical exam which effectively addresses the chief complaint and urgent patient issues
3. Disposition (PC7): Correctly assigns admitted patients to an appropriate level of care (ICU/Telemetry/Floor/ Observation Unit)
4. Systems-based Management (SBP2): Demonstrates the ability to call effectively on other resources in the system to provide optimal health care

You are welcome to bring in any resources you like including your smart phone.  Please do not give or receive any information about cases.

Thanks!
