# Supplementary material for: Epistaxis With Warfarin Coagulopathy: An Adult Simulation Case for Residents
Source: MedEdPORTAL. 2020 Jun 26;16:10916. doi: 10.15766/mep_2374-8265.10916 (PMC7331959; doi:10.15766/mep_2374-8265.10916)
Supplement: Supplementary file 1 — Simulation Case.docxSimulation Images.pptxPrebrief.docxDebriefing Materials.docxCritical Action Checklist.docxLearner Evaluation Form.docxHandout and Video Review.docx [file mep_2374-8265.10916-s001.zip › D. Debriefing Materials.docx]

**Epistaxis with Warfarin Coagulopathy**

**Debriefing Materials**

The focus of the simulation should be the learning during the debrief. This case lends itself to several debriefing methods. Use of the advocacy-inquiry methodology is preferred, but plus/delta or a combination of the two can be used successfully.

Potential debriefing questions to promote successful discussion:

1. How do you feel about the case?
2. In what areas did you do well as a team?
3. What are some areas of improvement in the future?
4. Do you feel you used closed-loop communication throughout the case?
5. What was wrong with the patient? Can you give a summary of the case?
6. Can you explain a step-wise approach to addressing epistaxis?
7. How did you choose to treat the coagulopathy? How might you treat coagulopathy differently in the future?
8. Why did the patient decompensate when the nasal packing was placed? (The blood clot was pushed into the oropharynx)
9. What is a take home point from today’s case?

**Epistaxis**

The most common location for bleeding in epistaxis is anterior and related to Kiesselbach’s plexus (watershed area). Posterior bleeding is usually related to the posterolateral branches of the sphenopalatine artery but may be related to the carotid artery.

The most common etiology of epistaxis is nose picking. Other causes include mucosal dryness (during winter months), mucosal hyperemia, foreign body, chronic excoriation (cocaine related), and trauma.

Several conditions place individuals at an increased risk of epistaxis. These include anticoagulation and antiplatelet therapy, hereditary hemorrhagic telangiectasia (Osler-Weber-Rendu), familial blood dyscrasias, carotid artery aneurysm, and the use of aspirin, alcohol, and cocaine.

The management of epistasis should follow a stepwise approach. First, direct pressure should be applied. Patients often quickly tire by pinching the nose. A nasal clamp should be immediately placed. Once the active bleeding is stopped, attention should be turned to preparing equipment and medications. Common equipment includes bayonet forceps, a nasal speculum and suction with a Yankauer catheter. A good light source will also be needed. Make sure to don appropriate personal protective equipment including glasses, a mask and a gown. Have the patient forcefully blow any clots from each nostril. This evacuates the clot prior to potential packing and allows for better visualization of the area of active bleeding. Failure to adequately perform this step may result in suboptimal visualization and medication penetration. Residual clots may be pushed into the pharynx potentially leading to choking or airway compromise.

The initial medication of choice is likely related to physician preference and immediate availability, but likely includes a vasoconstrictor such as Phenylephrine (Neo-Synephrine) or Oxymetazoline (Afrin) and an anesthetic such as topical lidocaine. This can be applied by spraying or by soaking a pledget and inserting it for around 5 minutes.

The next medication should likely be a thrombogenic agent. Tranexamic Acid (TXA) 500mg in 5mL applied to a pledget has been shown to reduce the likelihood of rebleeding compared to other medications. Other agents include Gelfoam, Surgicel, and FloSeal.

Cauterization may also be considered. Do not attempt chemical or electrical cauterization unless the area of bleeding is visualized. The area must be anesthetized first for patient comfort.

Definitive management of epistaxis may require placement of a nasal packing. Historically, this was done with gauze ribbon, but placement can be difficult. The ribbon can partially or fully dislodge and result in airway compromise. Newer inflatable balloon catheters now provide an easy and effective option. Rapid Rhino manufactures a nasal pack device that can be easily inserted. Soak the device briefly in sterile water. Use the 4.5-5.5cm device for anterior bleeding and 7.5cm for posterior bleeding.

Patients with controlled anterior epistaxis may be discharged after 1 hour of observation. They should follow up in 48-72 hours to have the packing removed. Admission in required for posterior packing or for bilateral anterior packing. Antibiotics are not indicated if the packing will be removed within 72 hours.

**Treatment of Serious Bleeding with Warfarin Coagulopathy**

Patients on warfarin with serious, life-threatening bleeding should be reversed regardless of their INR. Vitamin K 10mg IV should be given promptly. Prothrombin Complex Concentrate (PCC or Kcentra) should be given as a fixed dose or using manufacturer-recommended dosing based on patient weight and INR. If PCC is not available Fresh Frozen Plasma (FFP) may be used.

**Debrief Checklist**

- Reaction (How do you feel about the case?)
- Review critical actions
- Physical examination
  - Was an appropriately focused physical examination performed?
- Communication
  - Closed-loop
- Leadership and teamwork
  - Was there a clear leader?
  - How did the leader function?
- Emergency stabilization
  - Did you prioritize critical initial stabilization and reassess after stabilizing intervention?
- Epistaxis management
  - How did you choose to stop the bleeding?
  - What was (or would have been) your second method?
- Management of choking on blood clot
  - Why did the patient choke on the clot? (He was unable to blow the clot from the nose prior to placement of the packing)
  - How would management differ if this were an anterior bleed?
- Systems-based Management
  - Did you call effectively on other resources (consultants)?
- Why did the patient have a PEA arrest (only if suctioning was not performed)? (Hypoxia due to blood clot pushed into the pharynx)
- Management of warfarin-induced coagulopathy
  - How would management differ if the patient were using a Xa inhibitor?
- Disposition
  - Did you admit the patient to the appropriate level of care?
- Take home (What is something you learned today that you will apply in the future?)
