# Supplementary material for: Epistaxis With Warfarin Coagulopathy: An Adult Simulation Case for Residents
Source: MedEdPORTAL. 2020 Jun 26;16:10916. doi: 10.15766/mep_2374-8265.10916 (PMC7331959; doi:10.15766/mep_2374-8265.10916)
Supplement: Supplementary file 1 — Simulation Case.docxSimulation Images.pptxPrebrief.docxDebriefing Materials.docxCritical Action Checklist.docxLearner Evaluation Form.docxHandout and Video Review.docx [file mep_2374-8265.10916-s001.zip › E. Critical Action Checklist.docx]

**Epistaxis with Warfarin Coagulopathy**

**Critical Action Checklist**

Participants: ____________________________ Faculty: ________________________________

____________________________

____________________________

____________________________

| **Action** | **Action Complete** | | | **Notes** |
| --- | --- | --- | --- | --- |
|  | **Yes** | **Partially** | **No** |  |
| Immediately address epistaxis with compression follow with nasal packing or cautery |  |  |  |  |
| Treat acute blood-loss anemia and shock with blood products |  |  |  |  |
| Recognize mild hypertension and choose not to treat with antihypertensive medications in the setting of acute blood loss anemia |  |  |  |  |
| Treat warfarin-induced coagulopathy with appropriate reversal agent |  |  |  |  |
| Recognize airway obstruction and treat with suctioning |  |  |  |  |
| Communicate clearly with consultant(s) |  |  |  |  |
| Direct appropriate ICU admission |  |  |  |  |

Notes:
