# Supplementary material for: Epistaxis With Warfarin Coagulopathy: An Adult Simulation Case for Residents
Source: MedEdPORTAL. 2020 Jun 26;16:10916. doi: 10.15766/mep_2374-8265.10916 (PMC7331959; doi:10.15766/mep_2374-8265.10916)
Supplement: Supplementary file 1 — Simulation Case.docxSimulation Images.pptxPrebrief.docxDebriefing Materials.docxCritical Action Checklist.docxLearner Evaluation Form.docxHandout and Video Review.docx [file mep_2374-8265.10916-s001.zip › F. Learner Evaluation Form.docx]

**Epistaxis with Warfarin Coagulopathy**

**Learner Evaluation Form**

| 1 | 2 | 3 | 4 | 5 |
| --- | --- | --- | --- | --- |
| Strongly   Disagree | Disagree | Neither Agree or Disagree | Agree | Strongly  Agree |

**The session enhanced my medical knowledge and clinical skills:**

① ② ③ ④ ⑤

**The session will be helpful to me in my practice:**

① ② ③ ④ ⑤

**The session will help to improve my overall patient outcomes:**

① ② ③ ④ ⑤

**The session was realistic and clinically relevant:**

① ② ③ ④ ⑤

**The simulators/equipment/standardized patients utilized in the session were effective in allowing me to learn and perform the tasks:**

① ② ③ ④ ⑤

**The faculty were organized and prepared:**

① ② ③ ④ ⑤

**The staff was friendly and helpful:**

① ② ③ ④ ⑤

**What did you find most valuable about the session?**

**________________________________________________________________________________________________________________________________________________________________________________________________________________________________________________________________________________________________________________________________________________________________________________________________________________________________________________________________________________**

**What did you find least valuable about the session?**

**________________________________________________________________________________________________________________________________________________________________________________________________________________________________________________________________________________________________________________________________________________________________________________________________________________________________________________________________________________**

**Suggestions for improvement:**

**________________________________________________________________________________________________________________________________________________________________________________________________________________________________________________________________________________________________________________________________________________________________________________________________________________________________________________________________________________**
