# Supplementary material for: Epistaxis With Warfarin Coagulopathy: An Adult Simulation Case for Residents
Source: MedEdPORTAL. 2020 Jun 26;16:10916. doi: 10.15766/mep_2374-8265.10916 (PMC7331959; doi:10.15766/mep_2374-8265.10916)
Supplement: Supplementary file 1 — Simulation Case.docxSimulation Images.pptxPrebrief.docxDebriefing Materials.docxCritical Action Checklist.docxLearner Evaluation Form.docxHandout and Video Review.docx [file mep_2374-8265.10916-s001.zip › G. Handout and Video Review.docx]

**Epistaxis with Warfarin Coagulopathy**

**Handout**

**Epistaxis**

The most common location for bleeding in epistaxis is anterior and related to Kiesselbach’s plexus (watershed area). Posterior bleeding is usually related to the posterolateral branches of the sphenopalatine artery but may be related to the carotid artery.

The most common etiology of epistaxis is nose picking. Other causes include mucosal dryness (during winter months), mucosal hyperemia, foreign body, chronic excoriation (cocaine related), and trauma.

Several conditions place individuals at an increased risk of epistaxis. These include anticoagulation and antiplatelet therapy, hereditary hemorrhagic telangiectasia (Osler-Weber-Rendu), familial blood dyscrasias, carotid artery aneurysm, and the use of aspirin, alcohol, and cocaine.

The management of epistasis should follow a stepwise approach. First, direct pressure should be applied. Patients often quickly tire by pinching the nose. A nasal clamp should be immediately placed. Once the active bleeding is stopped, attention should be turned to preparing equipment and medications. Common equipment includes bayonet forceps, a nasal speculum and suction with a Yankauer catheter. A good light source will also be needed. Make sure to don appropriate personal protective equipment including glasses, a mask and a gown. Have the patient forcefully blow any clots from each nostril. This evacuates the clot prior to potential packing and allows for better visualization of the area of active bleeding. Failure to adequately perform this step may result in suboptimal visualization and medication penetration. Residual clots may be pushed into the pharynx potentially leading to choking or airway compromise.

The initial medication of choice is likely related to physician preference and immediate availability, but likely includes a vasoconstrictor such as Phenylephrine (Neo-Synephrine) or Oxymetazoline (Afrin) and an anesthetic such as topical lidocaine. This can be applied by spraying or by soaking a pledget and inserting it for around 5 minutes.

The next medication should likely be a thrombogenic agent. Tranexamic Acid (TXA) 500mg in 5mL applied to a pledget has been shown to reduce the likelihood of rebleeding compared to other medications. Other agents include Gelfoam, Surgicel, and FloSeal.

Cauterization may also be considered. Do not attempt chemical or electrical cauterization unless the area of bleeding is visualized. The area must be anesthetized first for patient comfort.

Definitive management of epistaxis may require placement of a nasal packing. Historically, this was done with gauze ribbon, but placement can be difficult. The ribbon can partially or fully dislodge and result in airway compromise. Newer inflatable balloon catheters now provide an easy and effective option. Rapid Rhino manufactures a nasal pack device that can be easily inserted. Soak the device briefly in sterile water. Use the 4.5-5.5cm device for anterior bleeding and 7.5cm for posterior bleeding.

Patients with controlled anterior epistaxis may be discharged after 1 hour of observation. They should follow up in 48-72 hours to have the packing removed. Admission in required for posterior packing or for bilateral anterior packing. Antibiotics are not indicated if the packing will be removed within 72 hours.

**Treatment of Serious Bleeding with Warfarin Coagulopathy**

Patients on warfarin with serious, life-threatening bleeding should be reversed regardless of their INR. Vitamin K 10mg IV should be given promptly. Prothrombin Complex Concentrate (PCC or Kcentra) should be given as a fixed dose or using manufacturer-recommended dosing based on patient weight and INR. If PCC is not available Fresh Frozen Plasma (FFP) may be used.

**Epistaxis with Warfarin Coagulopathy**

**Video Review Form**

After reviewing the video and handout, please answer the following questions.

What did you do well?

What can you do better next time?

What did you notice watching the video that you did not notice during the case?

What are your “take-aways” from this simulation case.
