# Supplementary material for: Epistaxis With Warfarin Coagulopathy: An Adult Simulation Case for Residents
Source: MedEdPORTAL. 2020 Jun 26;16:10916. doi: 10.15766/mep_2374-8265.10916 (PMC7331959; doi:10.15766/mep_2374-8265.10916)
Supplement: Supplementary file 1 — Simulation Case.docxSimulation Images.pptxPrebrief.docxDebriefing Materials.docxCritical Action Checklist.docxLearner Evaluation Form.docxHandout and Video Review.docx [file mep_2374-8265.10916-s001.zip › _Educational_Summary_Report_10916.pdf]

MedEdPORTAL® The AAMC Journal of Teaching and Learning Resources

Original Publication

Open Access

Epistaxis With Warfarin Coagulopathy: An Adult Simulation Case for Residents

Jonathon Deibel, MD*

*Corresponding author: deibe1jp@cmich.edu

Abstract

Introduction: Epistaxis is a common presenting complaint in the emergency department. Proper technique to control the bleeding is essential. Active bleeding on an anticoagulant requires special consideration. Blood products and coagulopathy reversal are an important part of the resuscitation of an unstable bleeding patient on warfarin. **Methods:** This resource was created to simulate a high-acuity and moderate-frequency event seen in emergency departments and on hospital wards. The target audience included emergency department residents, internal medicine residents, and advanced practice providers. The scenario detailed the case of an 82-year-old male on Coumadin who presented with epistaxis. A mannequin equipped with an epistaxis task trainer in which rate of bleeding could be controlled was required. The case was complicated by a choking episode on attempted nasal packing. It also involved warfarin coagulopathy requiring blood products and warfarin reversal. The simulation may be performed in a simulation lab or in situ in the emergency department, intensive care unit, or medical floor. Critical actions include addressing epistaxis with packing, recognizing blood-loss anemia related to warfarin coagulopathy, and recognizing and managing airway obstruction. **Results:** Approximately 35 learners completed this module in five separate sessions. Written evaluation from learners showed that 95% felt the simulation scenario and debriefing were effective. **Discussion:** Simulation is an ideal teaching tool for this life-threatening presentation. Learners can demonstrate proper technique and management of this difficult case.

Keywords
Simulation, Epistaxis, Warfarin, Coagulopathy, Emergency Medicine, ENT, Hospital Medicine, Otolaryngology, Surgery - General

Educational Objectives

By the end of this activity, learners will be able to:

1. Demonstrate appropriate management of a patient presenting with active epistaxis, including compression and packing or cautery.
2. Treat blood-loss anemia and coagulopathy appropriately.
3. Demonstrate effective leadership of a treatment team in the emergency department setting.

Introduction

Epistaxis is a common complaint in the emergency department, occurring in up to 60% of the general population.[1] Patients often attempt various methods to stop the bleeding at home. By the time of arrival, they may have anemia due to significant blood loss. Warfarin is a commonly prescribed for the management of patients with certain conditions, such as atrial fibrillation, deep vein thrombosis, pulmonary embolism, and so on. Not infrequently, patients on warfarin develop a supratherapeutic international normalized ratio (INR). This puts them at an increased risk of blood-loss anemia and other bleeding complications. Patients have an increased risk of bleeding with an INR $>$ 3 and a substantially increased risk with an INR $>$ 5.[2] Occasionally, a patient presents with both epistaxis and warfarin coagulopathy.

Although frequently seen in the emergency department, epistaxis has traditionally not been taught in the simulation lab as a task trainer is not generally available. This resource uses an epistaxis simulator, the creation of which has been described in several publications.[3,4]

The target audience includes residents in training. The field most likely to encounter this scenario would be emergency medicine. However, the scenario may also be seen in the fields of internal medicine, family medicine, surgery, and otolaryngology.

Citation:
Deibel J. Epistaxis with warfarin coagulopathy: an adult simulation case for residents. *MedEdPORTAL*. 2020;16:10916.
https://doi.org/10.15766/mep_2374-8265.10916

Copyright © 2020 Deibel. This is an open-access publication distributed under the terms of the Creative Commons Attribution license.

Review of the *MedEdPORTAL* literature demonstrates no similar cases. A case of a closed head injury patient on warfarin does discuss rapid reversal of coagulopathy.[5] General management of warfarin is also discussed.[6] No simulation cases for epistaxis are currently available in *MedEdPORTAL*. This novel resource provides a simulation case to address learning competency in both epistaxis and warfarin coagulopathy.

Methods

Development

This case was used in an emergency medicine residency training program and was developed to be used as part of a rotating 3-year simulation curriculum. Learners were grouped into teams of about five residents and one to two students. The teams chose their own leader, generally a second-year resident. The leader assigned roles, including obtaining history and physical exam, giving medications, and managing the airway, among others. The senior resident took the role of attending physician, intervening and making suggestions as necessary for patient safety. Junior residents and medical students filled team-member roles, including performing the physical exam, managing the airway, and performing procedures. Each resident participated once in his or her training during the first, second, or third year. The team generally comprised at least one resident from each year of training. This pattern of simulation learning was used monthly within this training program. The learners were provided with a prebrief for the case. This prebrief did not give any hints or medical knowledge for the case. It offered a tool for faculty to use to focus learners' performance on select skills such as communication, physical exam, and procedures.

Equipment/Environment

The mannequin for the case was male, able to speak, and equipped with an epistaxis task trainer. The epistaxis task trainer allowed for control of the bleeding by a simulated nurse in the room. The patient had active bleeding from the nose as learners entered the room. Bleeding did not stop until the nose was packed. The patient was not on a cardiac monitor and did not have any lines in place to start the case. Other equipment in the room included a cardiac monitor, IV, crash cart, O2 (nasal cannula, nonrebreather mask), airway equipment, suction, intranasal packing, nasal clamps, and electrical cautery. Medications that were available included all advanced cardiac life support medications, common antihypertensives (hydralazine, labetalol, clonidine), 2L of normal saline, packed red blood cells (PRBCs), vitamin K, prothrombin complex concentrate (PCC), fresh frozen plasma (FFP), intranasal tranexamic acid, oxymetazoline (Afrin), adrenaline, and silver nitrate.

Personnel

A single simulated nurse was present in the room. This individual assisted the learners in finding needed equipment and medications. The simulated nurse controlled the rate of bleeding for the epistaxis task trainer. From the control room, the faculty acted as the voice of the patient, specialist (otolaryngologist), and/or admitting physician. Over the phone, these individuals prompted learners to perform delayed critical actions.

Implementation

The case presentation was available in the simulation case file (Appendix A). Visual stimuli materials including the door note, chest X-ray, and lab results were found in the simulation images file (Appendix B). The case was video recorded for later review by the participants. A prebrief was available for distribution to learners before the case (Appendix C). Debriefing material and a critical action checklist were available to the facilitator (Appendices D and E). Learners could offer feedback about the case on the learner evaluation form (Appendix F). Learners were asked to review the video recording of their performance and to self-evaluate using a provided handout and video review form (Appendix G).

Assessment

Verbal and written feedback from the case was very positive. Written feedback was collected after completion of the case and debrief using a required survey on the New Innovations residency management software. The case was complex and unique, but the resident learners appreciated the usefulness of the information. They felt it could be applied to their practice.

Debriefing

The focus of the simulation was on the learning during the debrief. The debrief was done both as a group and individually. Formal, facilitated group debriefing occurred immediately following the case. This case lent itself to several debriefing methods. We used advocacy-inquiry methodology, but plus/delta or a combination of the two could be used successfully. Teaching points and a debriefing checklist were provided as part of the debriefing materials (Appendix E).

Potential debriefing questions to promote successful discussion included the following:

- How do you feel about the case?
- In what areas did you do well as a team?
- What are some areas of improvement in the future?
- Do you feel you used closed-loop communication throughout the case?

Copyright © 2020 Deibel. This is an open-access publication distributed under the terms of the Creative Commons Attribution license.

- What was wrong with the patient? Can you give a summary of the case?
- Can you explain a stepwise approach to addressing epistaxis?
- How did you choose to treat the coagulopathy? How might you treat coagulopathy differently in the future?
- Why did the patient decompensate when the nasal packing was placed? (The blood clot was pushed into the oropharynx.)
- What is a take-home point from today's case?

After completion of the case and facilitated debrief, the learners were provided with a handout of the key teaching points and were asked to complete a self-evaluation using the handout and video review form (Appendix G).

Results

Written evaluations showed that 95% of the learners felt the simulation scenario and debriefing were effective. Asked if the knowledge and skill gained from the training session would be helpful in practice, 59% of learners strongly agreed, and 41% agreed. The same percentages were seen when asked if the knowledge and skill learned in the session would help to improve their patient outcomes. Specifically, residents wrote that the simulation was "very well organized and engaging" and that the day was the "best of the year."

The case was managed and debriefed with one faculty member. This faculty member was trained in emergency medicine and built the case after two similar patient encounters.

Discussion

This simulation case requires significant faculty and simulation center preparation for successful implementation. It combines two common but rarely practiced presentations to an emergency department, namely, epistaxis and warfarin coagulopathy. The epistaxis task trainer must be created and the simulated nurse appropriately trained for the simulation to be successful. The primary challenge was planning for and adapting in real time to the complexity of the case. We were generally able to anticipate choices in medications and techniques. The scenario programming was created to flow in a fixed manner; however, the actual case implementations were mostly nonlinear because the teams timed the various interventions differently. Faculty used the various preprogrammed states and made on-the-fly adjustments to keep the case dynamic.

To set up the epistaxis trainer, a CPR mannequin was used with tubing that ran internally from the back of the mannequin to the posterior nose. The tubing was connected to a bag with simulation blood, whose rate of flow could be controlled by a roller clamp. The bag of simulated blood was covered to hide its contents. By using a CPR mannequin, some fidelity in the simulation was lost, requiring the simulated nurse to verbalize specific findings such as heart and lung sounds, pulses, and details of the intranasal and oral exams. Learners must demonstrate buy-in to the simulation as some of the fidelity is lost in creating an epistaxis task trainer.

As learners can manage the case successfully using multiple pathways, a multitude of medications, devices, and blood products must be available to them. Anticipated medications include phenylephrine, oxymetazoline, epinephrine, lidocaine, tranexamic acid, silver nitrate, vitamin K, and PCC. Devices that should be available include Gelfoam, Surgicel, FloSeal, ribbon gauze, pledgets, and nasal tamponade balloons of various sizes. Learners may choose to use PRBCs, FFP, or platelets. This large array of options required the simulation staff to obtain materials and medications for and become familiar with many new treatments. Piloting the scenario was helpful in ensuring that needed supplies were available and provided to the learners at the correct times.

The climax of the case is when the patient chokes on a blood clot. The learners have to recognize the blood clot as the cause of the choking and intervene appropriately. The simulated nurse must help the learners through this portion of the case as some fidelity is lost in that a blood clot is not actually used for learners to see. During the planning and piloting phase, several attempts to place a blood clot in the oropharynx during the case were unsuccessful. This challenge was overcome by having the simulated nurse verbalize that a large clot was present and able to be suctioned out if the resident looked for a clot or performed suctioning. Practice through a pilot case ensured the simulated nurse's proper timing and verbalization of the clot to learners.

The clinical scenario and medical management were new to the simulation staff, requiring some preliminary teaching and subsequent redirection by faculty. Once the simulation staff became familiar with the case, they tried several times to inappropriately influence the learners on their management. For example, the patient presented with hypertension but decompensated if treated with an antihypertensive. The simulated nurse brought the hypertension to the learners' attention and suggested they treat. The learners interpreted this suggestion as a prompt and incorrectly treated with an antihypertensive. Conversely, the simulated nurse suggested the administration of PRBCs too early in the scenario, depriving the

Copyright © 2020 Deibel. This is an open-access publication distributed under the terms of the Creative Commons Attribution license.

learners of the chance to make this choice on their own. These missteps and several similar others were remedied during the pilot or first actual case.

While time is needed to properly develop and pilot this scenario, this case has been well received. We plan to keep it in our regular 3-year rotation of cases to allow every resident to have this important experience during training.

Appendices

A. Simulation Case.docx

B. Simulation Images.pptx

C. Prebrief.docx

D. Debriefing Materials.docx

E. Critical Action Checklist.docx

F. Learner Evaluation Form.docx

G. Handout and Video Review.docx

All appendices are peer reviewed as integral parts of the Original Publication.

Jonathon Deibel, MD: Assistant Clinical Professor, Emergency Medicine, Central Michigan University College of Medicine

Disclosures

None to report.

Funding/Support

None to report.

Ethical Approval

Reported as not applicable.

References

1 Kucik CJ, Clenney T. Management of epistaxis. *Am Fam Physician*. 2005;71(2):305-311.

2. Torn M, van der Meer FJM, Rosendaal FR. Lowering the intensity of oral anticoagulant therapy: effects on the risk of hemorrhage and thromboembolism. *Arch Intern Med*. 2004;164(6):668-673. https://doi.org/10.1001/archinte.164.6.668

3. Scott GM, Roth K, Rotenberg B, Sommer DD, Sowerby L, Fung K. Evaluation of a novel high-fidelity epistaxis task trainer. *Laryngoscope*. 2016;126(7):1501-1503. https://doi.org/10.1002/lary.25652

4. Pettineo CM, Vozenilek JA, Kharasch M, Wang E, Aitchison P. Epistaxis simulator: an innovative design. *Simul Healthc*. 2008;3(4):239-241. https://doi.org/10.1097/SIH.0b013e31816fdd22

5. Kohls GS, Ten Eyck R. Head injury in an apple picker. *MedEdPORTAL*. 2011;7:9013. https://doi.org/10.15766/mep_2374-8265.9013

6. Graham J, Noble R, Thachil J. Warfarin management in practice. *MedEdPORTAL*. 2015;11:10234. https://doi.org/10.15766/mep_2374-8265.10234

Received: April 19, 2019
Accepted: December 10, 2019
Published: June 26, 2020

Copyright © 2020 Deibel. This is an open-access publication distributed under the terms of the Creative Commons Attribution license.
